# Tissue Damage Disrupts Developmental Progression and Ecdysteroid Biosynthesis in *Drosophila*

**DOI:** 10.1371/journal.pone.0049105

**Published:** 2012-11-13

**Authors:** Jennifer F. Hackney, Omid Zolali-Meybodi, Peter Cherbas

**Affiliations:** Department of Biology, Indiana University, Bloomington, Indiana, United States of America; University of Massachusetts Medical School, United States of America

## Abstract

In humans, chronic inflammation, severe injury, infection and disease can result in changes in steroid hormone titers and delayed onset of puberty; however the pathway by which this occurs remains largely unknown. Similarly, in insects injury to specific tissues can result in a global developmental delay (e.g. prolonged larval/pupal stages) often associated with decreased levels of ecdysone – a steroid hormone that regulates developmental transitions in insects. We use *Drosophila melanogaster* as a model to examine the pathway by which tissue injury disrupts developmental progression. Imaginal disc damage inflicted early in larval development triggers developmental delays while the effects are minimized in older larvae. We find that the switch in injury response (e.g. delay/no delay) is coincident with the mid-3rd instar transition – a developmental time-point that is characterized by widespread changes in gene expression and marks the initial steps of metamorphosis. Finally, we show that developmental delays induced by tissue damage are associated with decreased expression of genes involved in ecdysteroid synthesis and signaling.

## Introduction

Insects proceed through a series of precisely timed developmental transitions during their life cycle. In *Drosophila melanogaster*, after egg hatching, larvae progress through three instars that are separated by a molt during which the cuticle is shed and re-synthesized to accommodate continued growth [1], [2]. The third and final instar is followed by pupariation, the onset of the larval-pupal transition which is characterized by eversion of anterior spiracles, contraction of the larval body and tanning of the larval cuticle to form the puparium [3], [4]. It is within this protective casing that metamorphosis to the adult occurs [3], [4], [5]. The timing of these developmental transitions is influenced by nutritional and environmental cues and is regulated by systemic signals such as steroid hormones that direct coordinated developmental responses throughout the insect [6], [7], [8].

In insects, localized tissue damage is frequently associated with a systemic injury response resulting in delayed development (e.g. prolonged larval or pupal stages) [9], [10], [11], [12], [13], [14], [15], [16], [17], [18], [19], [20], [21]. In *Drosophila*, damage to imaginal (adult precursor) tissues via irradiation, induction of cell death clones, or localized activation of apoptosis causes a prolonged third larval instar [10], [11], [13], [14], [20], [21]. The mechanism by which localized tissue damage disrupts developmental progression is poorly understood but appears to involve a reduced systemic hemolymph ecdysteroid titer.

In *Drosophila*, as in most arthropods, the timing of developmental transitions is coordinated by a transient rise in the titer of the steroid hormone ecdysone (E) [22], [23]. Production and release of ecdysteroids is regulated by a small secreted neuropeptide known as prothoracicotropic hormone (Ptth) [24], [25], [26], [27], [28], [29], [30]. Ptth stimulates ecdysone synthesis, at least in part, by regulating transcription of a number of Halloween genes, a family of genes encoding cytochrome P450 enzymes that are required for ecdysone synthesis in the prothoracic gland cells of the ring gland [24], [25], [26], [27], [28], [29]. Ecdysone is released from the ring gland into the hemolymph and transported to peripheral tissues where it is converted to its active form, 20-hydroxyecdysone (20E), which binds to its receptor comprised of the Ecdysone receptor (EcR) and Ultraspiracle (Usp) [31], [32], [33]. EcR/Usp heterodimers bind to DNA and regulate transcription of target genes such as *Ecdysone-inducible proteins* −*71CD* (*Eip71CD*), −*74EF (Eip74EF*), −*75B (Eip75B)*, −*78C* (*Eip78C)*, and *Br* (*Broad*) leading to widespread physiological changes and developmental progression [34], [35], [36], [37], [38], [39], [40], [41], [42]. It has been suggested that imaginal disc damage triggers developmental delays, possibly by preventing the synthesis or release of ecdysone [17], [20], [21], [43]. The mechanism by which injury leads to decreased hemolymph ecdysteroid titers remains unclear but appears to involve delayed release of Ptth [9], [10], [20], [44], [45], [46]. The effects of tissue damage on other components required for ecdysone synthesis and signaling are less clear.

The effects of injury on developmental progression are dependent upon the developmental stage of the animal at the time injury is sustained [9], [10], [11], [13], [19]. Imaginal tissue damage induced by irradiation or genetic cell ablation only appears to retard pupariation when induced at or before an Injury Response Checkpoint (IRC) which is reached sometime during the second half of the third larval instar [10], [11], [13], [17], [20], [21], [47]. The exact time that the IRC is reached has not been clearly defined, however a number of studies in *Drosophila* and *Lepidoptera* (*Ephestia kuhniella* and *Lymantria dispar*) have demonstrated that tissue damage induced early in the last larval instar retards development while injury inflicted closer to pupariation time no longer affects developmental timing [10], [11], [13], [19], [20], [21], [48], [49]. In *Drosophila*, there are two additional critical developmental time points that are known to occur during the third larval instar. One of these critical periods is Critical Weight (CW), a size-assessment checkpoint reached early in the third instar, after which starvation no longer influences the time to pupariation [50]. The second critical period is the Mid-third Instar Transition (MIT), a developmental time point which marks the initial steps of metamorphosis, is associated with widespread changes in gene expression, and occurs during the middle of the third larval instar [51]. The possibility that the IRC corresponds with another critical developmental checkpoint (e.g. CW, MIT) has not been explored.

Here we examine the timing of the IRC and the mechanism by which localized tissue damage triggers developmental delays. We find that imaginal disc damage leads to delayed onset of the MIT, pupariation and adult eclosion. The effects of injury on developmental timing are minimized or absent closer to pupariation time and the switch from retardation to no response is coincident with the MIT. In addition, we find that tissue damage is associated with (1) reduced ecdysteroid titers, (2) decreased expression of most genes involved in ecdysteroid synthesis and signaling and (3) increased expression of *Ecdysone oxidase* (*Eo*), a gene involved in ecdysone catabolism. Together our data suggest that systemic injury response signals act on multiple targets to regulate ecdysteroid titers and ecdysone signaling pathway components.

## Results

### Timing of Developmental Transitions at 18°C

**Figure 1:**
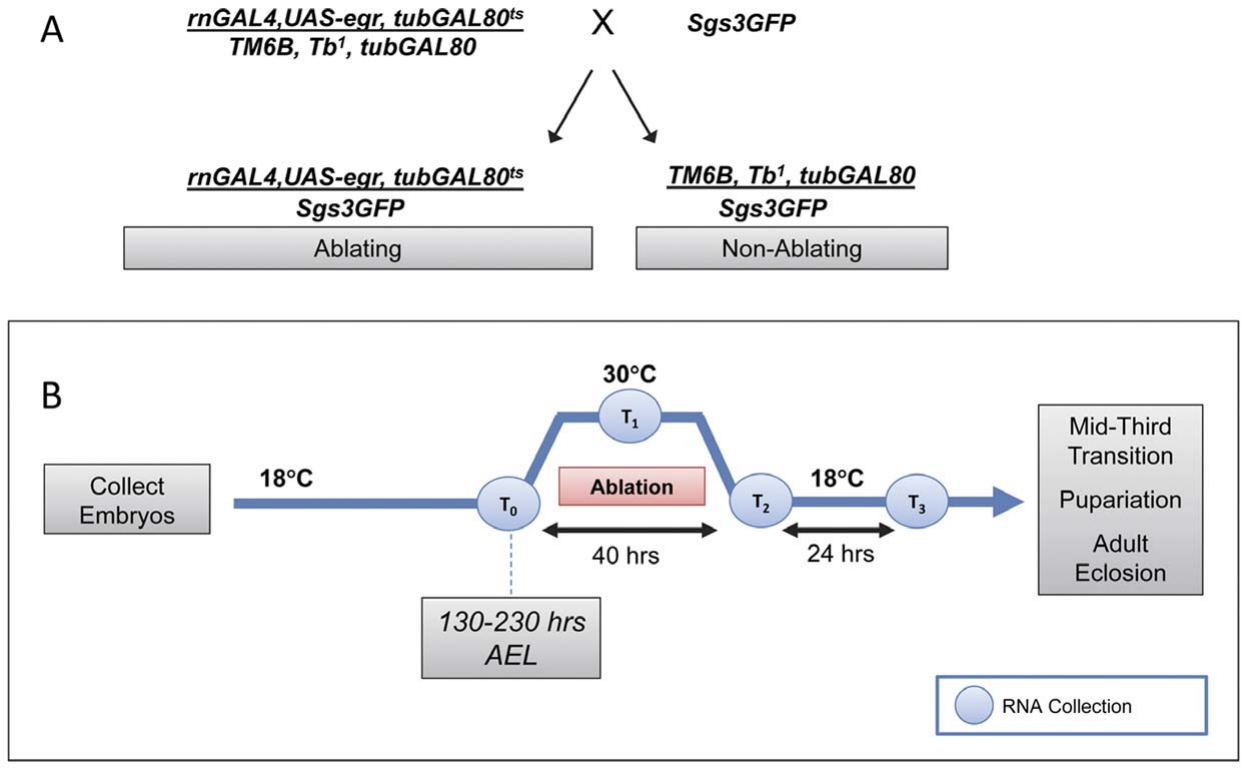
Cell Ablation Strategy. (A) Strategy used to produce Ablating and Non-Ablating larvae. w*; P{Sgs3-GFP}3 females were crossed to w*;+;rn-GAL4,UAS-egr,tubGAL80^ts^/TM6Tb, tubGAL80 males to give rise to the Ablating genotype (w*;+;rn-GAL4,UAS-egr,tubGAL80^ts^/Sgs3GFP) and the control Non-Ablating genotype (w*;+;TM6Tb, tubGAL80/Sgs3GFP). (B) Strategy to induce cell ablation. Embryos of the Ablating and Non-Ablating genotypes were collected at room temperature in four hour intervals and transferred to 18°C. First-instar larvae (48 hours AEL) were transferred to a vial containing standard cornmeal-yeast-agar medium and were allowed to develop at 18°C until the designated time for ablation induction. At the designated time during L3 (130–230 hours AEL) vials were transferred to 30°C for 40 hours, returned to 18°C and monitored daily to document the time to *Sgs3GFP* expression, pupariation or eclosion. RNA for qPCR and samples for EIA experiments were collected at time points T_0_–T_3_.

To induce tissue damage, we utilized flies containing a *rnGAL4* enhancer trap [52], a *UAS-eiger* transgene, and a temperature sensitive GAL80 variant driven by a tubulin promoter (*tubGAL80^ts^*), all recombined onto a single third chromosome (Figure 1A) [21]. The *rnGAL4* driver is expressed throughout the third larval instar in the wing pouch, the peripodial epithelium overlying the wing pouch, the haltere disc and a ring in the leg discs [21]. In addition, we observed low but detectable levels of *rnGAL4* expression in 1–3 cells in each salivary gland throughout the third larval instar. *Eiger* (*egr*) encodes the *Drosophila* ortholog of *tumor necrosis factor-alpha* (*TNFα*) and induces cell death via downstream activation of c-Jun N-terminal kinase (JNK) [53], [54]. The temperature sensitive variant of GAL80 represses GAL4 function at 18°C but not at 30°C [55] and was used to regulate *egr* expression. As shown in Figure 1A, Males of the genotype w*;rnGAL4, UAS-egr, tubGAL80^ts^/TM6 Tb^1^, tubGAL80 were crossed to *w^1118^;+;+* (not shown) or w*; P{Sgs3-GFP}3 females to produce w*;+;rn-GAL4,UAS-egr,tubGAL80^ts^/P{Sgs3GFP}3 (referred to as Ablating Genotype) and w*;+;TM6Tb^1^, tubGAL80/P{Sgs3GFP}3 (referred to as Non-Ablating Genotype).

**Figure 2:**
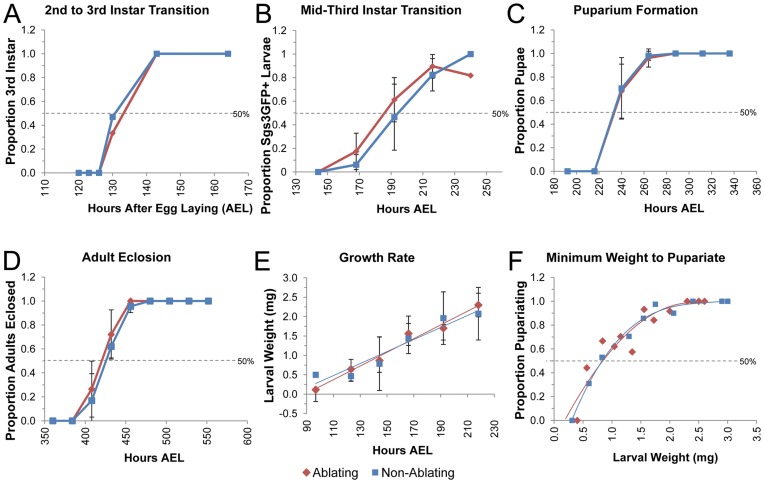
Timing of Developmental Transitions in Larvae Reared at 18 °C**.** (A–F) Ablating genotype (w*; rnGAL4, UAS-egr, tubGAL80^ts^/Sgs3GFP) is shown in Red. Non-Ablating genotype (w*; TM6, Tb^1^, tubGAL80/Sgs3GFP) is shown in Blue. All larvae were maintained at 18°C. (A) Fraction of larvae of the indicated genotype that had ecdysed to the 3^rd^ Larval Instar is plotted relative to time in hours After Egg Laying (AEL). (B) Fraction of larvae of the indicated genotype that had reached the mid-third transition (as measured by *Sgs3GFP* expression) is plotted relative to hours AEL. (C) Fraction of larvae of the indicated genotype that had undergone pupariation is plotted relative to hours AEL. (D) Fraction of larvae of the indicated genotype that had eclosed as adults is plotted relative to the time in hours AEL. (E) Plot of average larval weight (mg) at a given time after egg laying for Ablating and Non-Ablating larvae. (F) Fraction of larvae that underwent pupariation after starvation at a given size for Ablating and Non-Ablating larvae.

**Figure 3:**
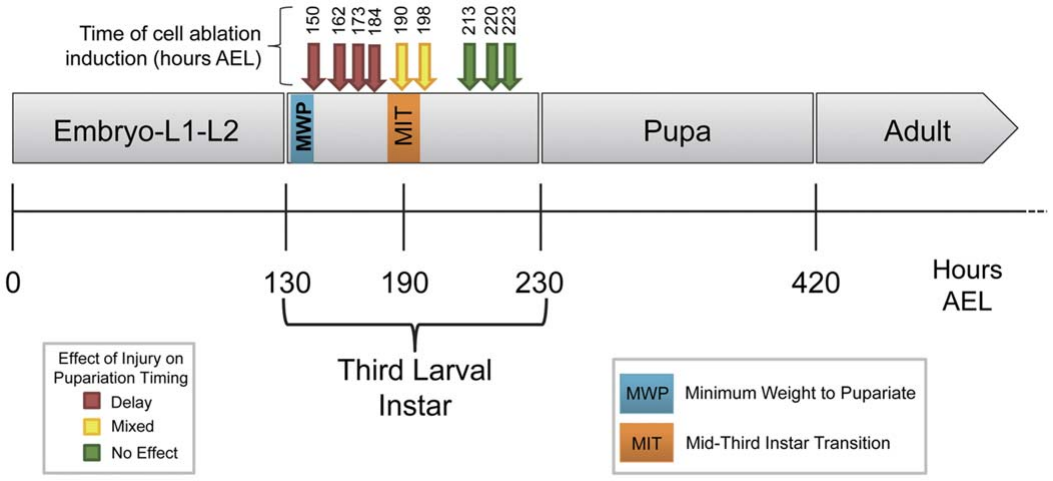
Switch in Injury Response Coincides with the Mid-third Instar Transition. Cell ablation was induced as described (Figure 1) at various time points (arrows) during the third larval instar. Cell ablation resulted in delayed pupariation (Red Arrows), no effect on developmental timing (Green Arrows), or a mixed effect (Yellow Arrows) in which some animals delayed development and others developed at the same time as controls.

The timing of developmental transitions is known to be influenced by temperature as well as genetic background [8], [56], [57]. Our first set of experiments was therefore designed to determine the timing of a number of developmental transitions for each of the genotypes (Ablating and Non-Ablating) when maintained at a constant temperature of 18°C, a temperature at which GAL80^ts^ inhibits GAL4 thereby preventing eiger-induced cell death. The molt from 2^nd^ (L2) to 3^rd^ larval instar (L3) was determined by examination of larval mouth hooks in animals reared at 18°C (Figure 2A). Larvae of the Ablating (n = 59) and Non-Ablating (n = 63) genotype molted to L3 at a similar time, approximately 130 hours after egg laying (AEL; Figure 2A, Figure 3).

The Mid-third Instar Transition (MIT) is a developmental time point associated with a low titer ecdysteroid pulse and is characterized by widespread changes in gene expression including induction of a glue gene – *Salivary gland secretion 3* (*Sgs3*) [51], [58]. The timing of the MIT was determined by examination of an *Sgs3GFP* reporter that is expressed in salivary glands beginning at the MIT [58], [59]. As shown in Figure 2B, there was no significant difference in the timing of onset of *Sgs3GFP* expression between Ablating (n = 250) and Non-Ablating (n = 192) genotypes when maintained at 18°C. Based on visualization of GFP in the salivary glands of whole larvae, the MIT appears to occur between 185 and 195 hours AEL (Figure 2B, Figure 3).

Larvae were maintained at 18°C and checked at intervals of 24 hours for completion of puparium formation (Figure 2C) and adult eclosion (Figure 2D). Pupariation time (time from midpoint of the egg-laying period to completion of puparium formation) was approximately 235 hours AEL for both Ablating (n = 42) and Non-Ablating (n = 44) genotypes (Figure 2C, Figure 3). Similarly, we found no significant difference in time to adult eclosion between Ablating and Non-Ablating animals. Adult eclosion occurred at approximately 420 and 425 hours AEL for Ablating (n = 37) and Non-Ablating (n = 42) genotypes, respectively (Figure 2D, Figure 3).

Critical Weight (CW) is the weight at which starvation no longer delays time to pupariation [50]. A second size assessment checkpoint is Minimum Viable Weight (MVW) which represents the weight at which larvae have enough nutritional stores in the form of fat body to survive the next developmental transition [50]. In *Drosophila*, CW and MVW occur at approximately the same time, early in the third larval instar [60], [61]. To identify the time that these checkpoints are reached in Ablating and Non-Ablating genotypes when maintained at 18°C, we determined the minimum weight needed for larvae to pupariate following starvation (Minimum Weight to Pupariate, MWP; Figure 2F). Third instar larvae of known weights were starved and the proportion of larvae that successfully pupariated was measured. Larvae of both the Ablating and Non-Ablating genotypes exhibited a 50% threshold for pupariation after starvation at approximately 0.9 mg/larva. Based on the growth rate observed during L3 (Figure 2E), larvae of both the Ablating (n = 199) and Non-Ablating (n = 200) genotypes are predicted to reach the CW/MVW checkpoint at approximately 142 hours AEL (MWP, Figure 3).

### Effects of Tissue Damage on the Mid-Third Instar Transition

**Figure 4:**
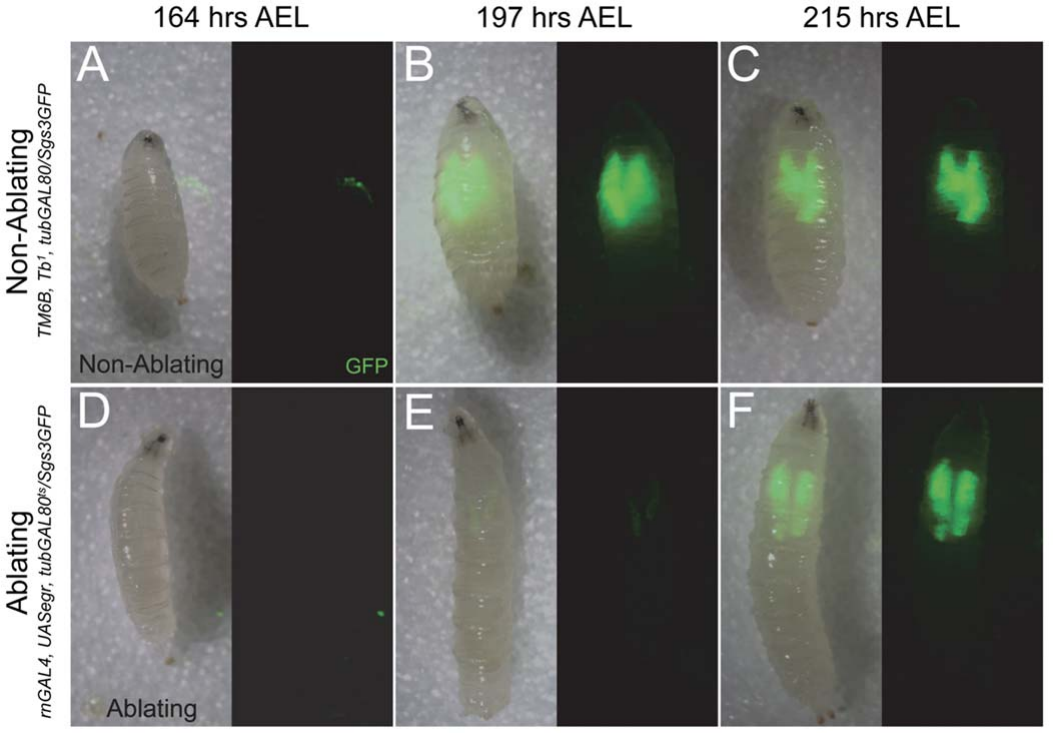
Imaginal Disc Damage Delays the Mid-Third Instar Transition. (A–F) Timing of the mid-third instar transition (as measured by *Sgs3GFP* expression) in larvae that were heat-treated for 24 hours at 172 hours AEL to induce tissue damage in wing discs. (A–C) *Sgs3GFP* expression in Non-Ablating (w*; TM6, Tb^1^, tubGAL80/Sgs3GFP) larvae. (A) *Sgs3GFP* expression is absent in most larvae at 164 hours AEL, just before heat treatment (Sgs3GFP^+^ = 3.6±4.7%; n = 28). *Sgs3GFP* is expressed at high levels after heat treatment in salivary glands at (B) 197 hours AEL (Sgs3GFP^+^ = 77.3±0.6%; n = 22), at (C) 215 hours AEL (Sgs3GFP^+^ = 91.7±7.9%; n = 12) and at 236 hours AEL (Sgs3GFP^+^ = 100±0.0%; n = 19) – data not shown. (D–F) *Sgs3GFP* expression in Ablating (w*; rnGAL4, UAS-egr, tubGAL80^ts^/Sgs3GFP) larvae. (D) *Sgs3GFP* expression is absent in most larvae at 164 hours AEL, just before heat treatment (Sgs3GFP^+^ = 2.6±3.1%; n = 38). (E) *Sgs3GFP* expression is detected at low levels at 197 hours AEL in only a small fraction of larvae examined (Sgs3GFP^+^ = 10.0±13.3%; n = 30). (F) High levels of *Sgs3GFP* expression is visible at 215 hours AEL (Sgs3GFP^+^ = 53.9±37.9%; n = 26) and at 236 hours AEL (Sgs3GFP^+^ = 75.0±1.3%; n = 28) – data not shown.

To examine how localized tissue damage influences the timing of the MIT, we induced cell ablation in the wing imaginal discs (Figure 1B) at 172 hours AEL and examined larvae for expression of the *Sgs3GFP* reporter in salivary glands (Figure 4). At 164 hours AEL, before the induction of cell ablation, *Sgs3GFP* expression was observed in 3.6% of Non-Ablating larvae (n = 28; Figure 4A) and 2.6% of Ablating larvae (n = 38; Figure 4D). Following the heat-treatment to induce cell death via *eiger* expression in the wing discs, most (77.3%; n = 22) larvae of the Non-Ablating genotype expressed *Sgs3GFP* by 197 hours AEL (Figure 4B). *Sgs3GFP* expression was maintained at high levels throughout the remainder of the third larval instar and was detected in 91.7% of larvae at 215 hours AEL (n = 26; Figure 4C) and in 100% of larvae at 236 hours AEL (n = 19; data not shown) in Non-Ablating animals. In contrast, following induction of cell death, only 10.0% (n = 30) of Ablating larvae displayed any *Sgs3GFP* expression by 197 hours AEL and expression was consistently lower in Ablating larvae compared to Non-Ablating controls at this time point (compare figure 4E to 4B). High levels of *Sgs3GFP* expression were detected in only 53.9% (n = 26) of Ablating larvae by 215 hours AEL (Figure 4F) and in only 75.0% (n = 28) of larvae by 236 hours AEL (data not shown). We detected no obvious morphological defects in salivary glands following cell ablation and no signs of cell death within salivary glands at any time following cell ablation suggesting that delayed onset of *Sgs3GFP* expression is a result of imaginal disc cell ablation (data not shown).

### Influence of Tissue Damage on Time to Pupariation and Eclosion

**Figure 5:**
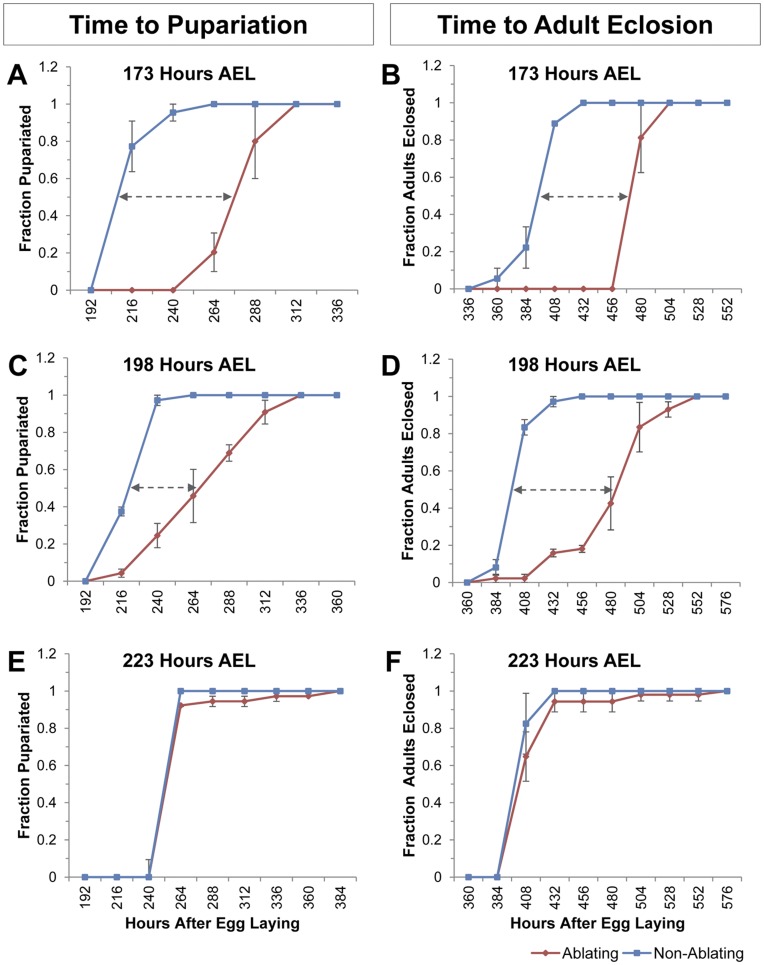
Damage to Wing Imaginal Discs Delays Pupariation and Adult Eclosion. (A–F) Timing of pupariation and adult eclosion following induction of cell ablation in the wing disc at the indicated time. Fraction of larvae that had (A, C, E) pupariated or (B, D, F) eclosed as adults are plotted relative to time in hours AEL for Ablating (Red - w*; rnGAL4, UAS-egr, tubGAL80^ts^/Sgs3GFP) and Non-Ablating (Blue - w*; TM6, Tb^1^, tubGAL80/Sgs3GFP) larvae. n = 3 independent populations (30 larvae each) were assayed for each ablation time. (A–B) Timing of (A) pupariation and (B) adult eclosion for larvae heat-treated at 173 hours AEL. Mean pupariation times are 286 and 227 hours AEL for Ablating and Non-ablating larvae, respectively. Mean eclosion times are 504 and 440 hours AEL for Ablating and Non-ablating larvae, respectively. Similar results were obtained with larvae heat-treated at 150, 162, or 184 hours AEL (data not shown). (C–D) Timing of (C) pupariation and (D) adult eclosion for larvae heat-treated at 198 hours AEL. Mean pupariation times are 279 and 230 hours AEL for ablating and non-ablating larvae, respectively. Mean eclosion times are 488 and 409 hours AEL for ablating and non-ablating larvae, respectively. Similar results were obtained with larvae heat-treated at 190 hours AEL (data not shown). (E-F) Timing of (E) pupariation and (F) adult eclosion for larvae heat-treated at 223 hours AEL. Mean pupariation times are 263 and 265 hours AEL for ablating and non-ablating larvae, respectively. Mean eclosion times are 419 and 411 hours AEL for ablating and non-ablating larvae, respectively. Similar results were obtained with larvae heat-treated at 213 and 220 hours AEL (data not shown).

Delay of pupariation was measured as the difference between mean pupariation time of Ablating larvae and Non-Ablating larvae housed in the same vial. To examine how localized tissue damage influences the timing of pupariation we induced cell ablation in the wing imaginal discs at 173 hours AEL, and then monitored the time to pupariation (Figure 5A). Cell ablation in the wing discs delayed pupariation by 59 hours (p<0.0001; Figure 5A). Similarly, adult eclosion was delayed by 64 hours (p<0.0001; Figure 5B) following the induction of cell death in imaginal discs at 173 hrs AEL.

### Influence of Larval Age on the Systemic Injury Response

To assess the effects of larval age on the systemic injury response we induced tissue damage in the wing imaginal discs in larvae of various ages. Cell ablation in the wing disc at 173 hours AEL delayed pupariation and adult eclosion by 59 and 64 hours, respectively (Figure 5A, B). Similar results were obtained when cell ablation was induced at 150, 162 or 184 hours AEL (Figure 3). Wing disc cell ablation induced at 198 hours AEL delayed mean pupariation and adult eclosion times by 49 hours (p<0.0001) and 79 hours (p<0.0001), respectively (Figure 5C, D). Similar results were obtained when cell ablation was induced at 190 hrs AEL (Figure 3). Injury induced between 190–198 hrs AEL resulted in two groups of Ablating larvae – those that delayed development in response to tissue damage and those that developed at the same time as Non-Ablating controls (See Figure 5D). Larvae that delayed development in response to wing disc ablation typically eclosed as adults with regenerated wings while those that eclosed at the same time as Non-Ablating controls emerged as wingless adults (Figure S1).

Wing disc cell ablation induced between 213–223 hours AEL resulted in no significant difference in the mean time to pupariation or adult eclosion in Ablating animals compared to Non-Ablating controls (Figure 5E, 5F; Figure 3). None of the Ablating animals showed any evidence of tissue regeneration; all emerged as wingless adults (Figure S1).

### Ecdysteroid Titers Following Tissue Damage

**Figure 6:**
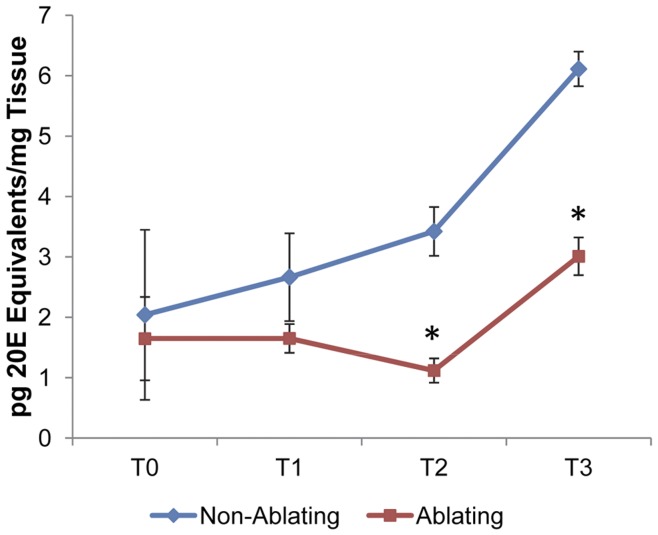
Ecdysteroid Titers Following Wing Disc Cell Ablation. Ecdysteroid titers measured for larvae of Ablating (Red) and Non-Ablating (Blue) genotypes at time points T_0_–T_3_ (See Figure 1B), as determined by EIA. Values are expressed as the means of 20E equivalents per mg of tissue. Error bars indicate the SEM (n = 2 samples of 15 larvae each). Asterisks indicate differences statistically significant at p≤0.05 (t-test).

The developmental retardation observed following imaginal disc cell ablation suggested the presence of an underlying ecdysteroid deficiency in injured animals. To measure the ecdysteroid titers in Ablating and Non-Ablating larvae, we performed an enzyme immunoassay (EIA) utilizing an ecdysteroid antiserum (Cayman Chemical). We examined ecdysteroid levels at four time points (Figure 1B): (T_0_) 170 hrs AEL - immediately before cell ablation was induced, (T_1_) 190 hrs AEL - half-way through the cell ablation period, (T_2_) 210 hrs AEL - immediately after the completion of cell ablation, and (T_3_) 234 hrs AEL –24 hours after cell ablation treatment was complete. As shown in Figure 6, just prior to the induction of cell ablation (T_0_) there was no significant difference in ecdysteroid titers between Ablating (1.65±0.98 pg 20E Equivalents/mg tissue) and Non-Ablating (2.04±1.99 pg 20E equiv/mg tissue) larvae. At T_1_ we detected a small (not statistically significant) difference between ecdysteroid concentrations in Ablating and Non-Ablating larvae; ecdysteroid concentrations were 1.65±0.34 and 2.66±1.03 pg 20E equivalents/mg tissue for larvae of the Ablating and Non-Ablating genotypes, respectively. Following the cell ablation period, ecdysteroid levels were significantly reduced (p<0.05) in Ablating larvae compared to their sibling Non-Ablating controls. At T_2_, ecdysteroid concentrations were 1.12±0.28 and 3.42±0.57 pg 20E equivalents/mg tissue for Ablating and Non-Ablating larvae, respectively while at T_3_ we detected 3.01±0.44 and 6.11±0.040 pg 20E equivalents/mg tissue for Ablating and Non-Ablating larvae, respectively.

### Effects of Tissue damage on Ecdysteroid Biosynthesis

To examine the effects of tissue damage on ecdysteroid signaling, we used qRT-PCR to examine injury-induced changes in expression of genes involved in ecdysone synthesis and signaling. Total RNA was isolated from Ablating and Non-Ablating larvae at time points T_0_–T_3_ (Figure 1B). For each genotype (Ablating and Non-Ablating), transcript levels in larvae at each time point (T_1_–T_3_) were compared to transcript levels in larvae at T_0_ to determine relative changes in gene expression.

**Figure 7:**
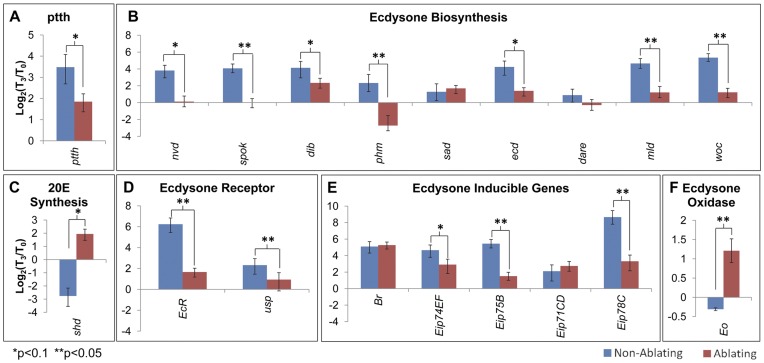
Effects of Tissue Damage on Ecdysone Biosynthesis and Signaling. qRT-PCR analysis of transcript levels of (A) *ptth*, (B) genes required for ecdysone synthesis, (C) *shd*, (D) ecdysone inducible genes, (E) ecdysone receptor components and (F) *ecdysone oxidase*. Graphs show changes in transcript levels 24 hours after heat treatment (T_3_; Figure 2B) compared to transcript levels immediately before heat treatment (T_0_; Figure 2B). Ablating (Red - w*/w^1118^; rnGAL4, UAS-egr, tubGAL80ts/+). Non-Ablating (Blue - w*/w^1118^; TM6, Tb^1^, tubGAL80/+).

To assess how tissue damage influences ecdysone synthesis, we examined expression of genes including (1) *ptth,* which encodes the neuropeptide that stimulates ecdysone synthesis in the ring gland [30], (2) genes encoding enzymes required for ecdysone synthesis in the ring gland including *neverland* (*nvd*) [62], *spookier* (*spok*) [63], *disembodied (dib)* [64], *phantom*(*phm*) [65], and *shadow* (*sad*) [66], and (3) genes encoding additional components required for ecdysone synthesis including *ecdysoneless* (*ecd*) [67], *Drosophila adrenodoxin reductase* (*dare*) [68], and transcription factors *molting defective* (*mld*) [69] and *without children* (*woc*) [70]. Following cell ablation in the wing disc (T_2_ and T_3_), we observed decreased expression of most ecdysteroidogenic genes in Ablating samples compared to Non-Ablating controls (Figure 7A-B, T_3_ Shown; Figure S2).

Conversion of ecdysone to 20-hydroxyecdysone is catalyzed by the Cyp450 enzyme encoded by *shade* (*shd*), which is expressed in peripheral tissues [71]. Following cell ablation in the wing disc, we observed increased *shd* expression in Ablating larvae compared to Non-Ablating controls (Figure 7C, T_3_ Shown; Figure S3).

### Effects of Injury on Ecdysone Signaling

The functional ecdysone receptor is comprised of a heterodimer formed by the Ecdysone Receptor (EcR) and the RXR homolog encoded by *ultraspiracle* (*usp*) [31], [32], [33]. Lower levels of expression were observed for both *EcR* and *usp* in Ablating larvae compared to Non-Ablating Controls following cell ablation in the wing imaginal disc (Figure 7D, T_3_ Shown; Figure S4).

To further examine the effects of injury on ecdysone signaling, we examined expression of ecdysone inducible genes including *Broad* (*br*) [41], [42], *Eip74EF* [39], *Eip75B* [40], *Eip71CD* [38], and *Eip78C* [37]. Expression of *Eip75B,* Eip74EF, and *Eip78C* were significantly reduced in Ablating larvae compared to Non-Ablating controls following cell ablation at time points T_2_ and T_3_ (Figure 7E, T_3_ Shown; Figure S5). There was no significant effect on tissue damage observed for *br* or *Eip71CD* (Figure 7E, T_3_ Shown; Figure S5).

### Ecdysone Catabolism Following Localized Tissue Damage

Ecdysone oxidase (Eo) is an enzyme that catalyzes the conversion of ecdysteroids into inactive 3-dehydroecdysteroids [72]. This ecdysteroid inactivation results in decreased ecdysteroid titers and helps to regulate the sharp ecdysteroid peaks that trigger developmental transitions. Following cell ablation in the wing disc (time points T_2_ and T_3_), *Eo* expression was elevated in Ablating samples compared to Non-ablating controls (Figure 7F, T_3_ Shown; Figure S3).

### Early Response to Injury

**Figure 8:**
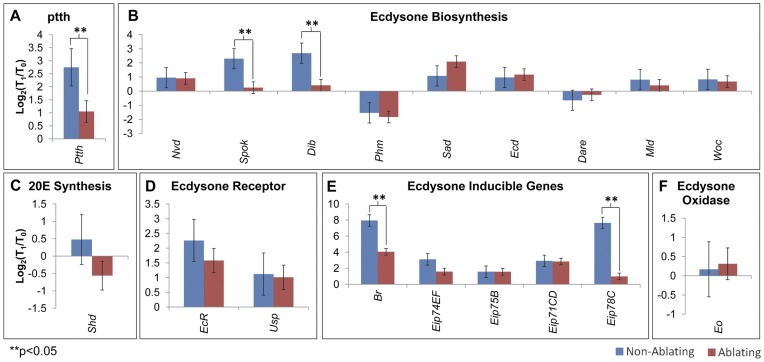
Early Response to Tissue Damage. qRT-PCR analysis of transcript levels of (A) *ptth*, (B) genes required for ecdysone synthesis, (C) *shd*, (D)ecdysone inducible genes, (E) ecdysone receptor components and (F) *ecdysone oxidase*. Graphs show changes in transcript levels mid-way through heat treatment (T_1_; Figure 2B) compared to transcript levels immediately before heat treatment (T_0_; Figure 2B). Ablating (Red - w*/w^1118^; rnGAL4, UAS-egr, tubGAL80ts/+). Non-Ablating (Blue - w*/w^1118^; TM6, Tb^1^, tubGAL80/+).

To identify potential differences between the early and late response to injury we examined expression of genes involved in ecdysteroid synthesis and signaling at an earlier time point, half-way through the cell ablation treatment (T_1_; Figure 1B). There was no significant difference for most genes examined in Ablating samples compared to Non-Ablating controls at T_1_ (Figure 8). Only five genes displayed reduced levels of expression in Ablating larvae compared to Non-Ablating controls at this early time point. At T_1_, ablating samples displayed significantly lower levels of expression of *ptth, spok, dib, Br, and Eip78C* compared to Non-Ablating controls (Figure 8).

## Discussion

Previous studies have indicated that injury to imaginal tissues is associated with prolonged larval and pupal stages but the effects of injury on developmental timing is minimized or even reversed in older larvae, after the animal has passed an Injury Response Checkpoint (IRC) [10], [17], [20], [47]. We find that cell ablation in wing imaginal discs delays all subsequent developmental transitions including the MIT (Figure 4), pupariation (Figure 5A), and adult eclosion (Figure 5B). We demonstrate for the first time that once larvae have progressed through the MIT, a time point that marks the initial steps of metamorphosis, injury no longer results in developmental retardation (Figure 3, 5,). These data suggest that the IRC coincides with the MIT and that events initiated at the onset of metamorphosis inhibit components of the systemic injury response.

Following wing disc cell ablation, the damaged tissues produce signals that retard development, thus providing time for imaginal tissue regeneration to occur [20], [21], [73], [74]. Tissue regeneration is inhibited if tissue damage is inflicted following the IRC (Figure S1) [20], [21]. It is possible that one or more of the genes that are up-regulated at the MIT may act to inhibit the injury response signals that mediate tissue regeneration and developmental retardation; however, interactions between injury response components, genes required for tissue regeneration, and genes that are differentially regulated at the MIT have not been explored.

Injury induced developmental delays are characterized by decreased hemolymph ecdysteroid titers. Halme et al [20] demonstrated that injury induced via x-irradiation triggers decreased expression of *ptth*, which encodes the cerebral neuropeptide that stimulates ecdysone synthesis in the ring gland. Similarly, here we have shown that targeted cell ablation in the wing imaginal disc leads to decreased expression of *ptth* and many genes required for ecdysone synthesis and signaling. Ptth has been shown to regulate expression of a number of genes required for ecdysone synthesis including *nvd*, *spok*, *phm* and *dib*, but it does not appear to directly regulate the expression of *sad* or *shd* [24]. We find that tissue damage is associated with decreased expression of ecdysteroidogenic genes with the exception of *sad* in the ring gland, and *shd* in peripheral tissues. These results are consistent with a model that injury produces signals that trigger decreased *ptth* expression, which in turn leads to transcriptional down-regulation of ecdysteroidogenic genes and subsequent decreases in ecdysone signaling components. It must be noted that ecdysteroidogenic genes can be regulated at both the transcriptional and post-translational level in response to signals other than Ptth including nutritional cues and insulin signaling [30]. A recent study has identified dILP8, a *Drosophila* insulin-like peptide that is produced by damaged imaginal tissues and mediates developmental delays [74]. Garelli et al (2012) demonstrated that dILP8 influences expression of both *dib* and *phm* but has no effect on *ptth* expression [74]. Together, these studies indicate that multiple factors likely participate in mediating the injury response.

Based on the observation that most ecdysteroidogenic genes are decreased following cell ablation in the wing disc, it is likely that the reduction in the ecdysteroid titer is due to decreased ecdysone synthesis in the ring gland. Our data indicate that decreased ecdysone titers may also result from enhanced ecdysteroid inactivation (Figure 7F). We observed an increase in expression of *ecdysone oxidase* – an enzyme that catalyzes the oxidation of ecdysteroids - following tissue damage [72]. This suggests the presence of multiple mechanisms that act in concert to reduce circulating ecdysteroid levels following injury.

Ecdysone response genes are largely down regulated in response to tissue damage. Two exceptions are *Eip71CD* and *br* (Figure 7E). *Eip71CD* and *br* each show tissue specific responses to ecdysone and are induced in response to ecdysone in some tissues and repressed by ecdysone in other tissues [51]. Our observations likely represent the combined effects of tissue-specific responses of these genes to ecdysone.

To assess the early effects of injury we analyzed changes in gene expression mid-way through the cell ablation procedure (T_1_, Figure 1B). At this early time point, we found that most ecdysteroidogenic genes were not yet affected (Figure 8). In contrast, five genes (*ptth*, *spok, dib*, *br*, *Eip78C*) displayed reduced expression in Ablating samples compared to Non-Ablating controls at T_1_. It is possible that these genes represent direct targets of the injury response and that systemic injury signals act directly on (1) the CNS to regulate *ptth* levels, (2) prothoracic glands to influence expression of key ecdysteroidogenic enzymes and (3) peripheral tissues to influence the expression of genes like *br*, which are essential during ecdysone signaling for the induction of early primary response genes [75].

## Materials and Methods

### Drosophila Stocks

w*;rnGAL4, UAS-egr, tubGAL80^ts^/TM6 Tb^1^, tubGAL80 [21] was a generous gift from I. Hariharan. w*; P{Sgs3GFP}3 [58], which expresses an Sgs3GFP fusion under the control of Sgs3 in an otherwise wild-type (Canton-S) background, and *w^1118^* were obtained from the Bloomington Stock Center. Unless otherwise indicated, flies were maintained at 22–25°C on a standard cornmeal-yeast-agar medium (Bloomington recipe).

### Developmental Timing Measurements

Fertilized eggs were collected at room temperature on grape juice agar plates. Collections were done in four hour intervals after which plates were transferred to 18°C. First instar larvae were collected from these plates 48 hours after egg laying and transferred (in groups of 30) to vials containing standard cornmeal-yeast-agar medium. Larvae were maintained at 18°C. For developmental progression, larvae were scored in 24 hour intervals. Larval stages were determined by observing mouth hook morphology. Pupariation was determined by observing contraction of larval body, eversion of spiracles, and onset of pigmentation of the puparium. Mean developmental times were tested for significant differences via two-sample t-test.

**Table 1:** Primers Used for qPCR.

	Target Gene	Fwd Primer	Rev Primer
**Ptth**	*ptth*	5′-TGGTGACCACCAAACGCAATGATG-3′	5′-ATCAGAAAGCGAGGGAAGTGCAGA-3′
**Ecdysone Synthesis**	*dare*	5′-ATCTAGTTGCGTGGATACGGGCAT-3′	5′-AGCCAGCCAGCTACATAAAGTCCA-3′
	*dib*	5′-AGTGGATGGAGTGACCAAGG-3′	5′-ACGAGCTCCAAAGGTAAGCA-3′
	*ecd*	5′-AGCGACTCGGATGAGTGGTTGAAT-3′	5′-GGCATTCATTTGTCCGTTCGGCTT-3′
	*nvd*	5′-AGCAACTTGTGTGTCATGCTTGGG-3′	5′-TTGGCTCCTAGGTGAGGGCAATAA-3′
	*phm*	5′-TGGGAAACCAAGAAGCTGAC-3′	5′-CGATTTCCTCCTGCTCTCAC-3′
	*sad*	5′-CAACGGGGACTGTTCTTCAT-3′	5′-CAGTGCGTCTTTTCCACTGA-3′
	*shd*	5′-GCACGAGGTATATGCGGATT-3′	5′-GGAGGTCGGAATGGGTATTT-3′
	*spok*	5′-GCTCTTTGGCGGTGATCGAAACAA-3′	5′-CGCCGAGCTAAATTTCTCCGCTTT-3′
	*mld*	5′-ACTGTGCGAACGGAATTGAACAGC-3′	5′-TGAGGATGCCATTGAGTGTGGTCT-3′
	*woc*	5′-ATCCCTGCTTCTCCGCCTTTAAGT-3′	5′-AGAAGACCTTCGGTGACTGCTGTT-3′
**Ecdysone Catabolism**	*Eo*	5′-AAGACCTACTCTCGCCTGCAACAA-3′	5′-TGTTTCATCCGTGGTACACCCAGT-3′
**Ecdysone Receptor**	*EcR-RA*	5′-ATATGTAGCTGTGCGTGGGTGTGT-3′	5′-AAGACTCCTATGCTGCAACCTCCA-3′
	*EcR-RE*	5′-TAGACGATGCACTTGCACTGTGG-3′	5′-ACATGTAGTTCTCCCTGTCTTTATAGC-3′
	*Usp*	5′-CCTGTGCCAAGTGGTCAACAAACA-3′	5′-ATCCAAGCGGCTTTCAGCAGAATC-3′
**Ecdysone Response**	*eip74EF*	5′-TTTCATCAAGTGGACGAACCGGGA-3′	5′-CATGTCCGGCTTGTTCTTGTGCAT-3′
	*eip75B*	5′-ATTGGATCAGGCGGCTCTTCTTCT-3′	5′-TGCTGCTGATGGTGCATATTGCTG-3′
	*eip71CD*	5′-ACGGAGGTGCTGGAAATCGACTAT-3′	5′-TGGTCAGGCCATACTCATGGTTGT-3′
	*eip78C*	5′-ATGTAAGCGGCGTACGTGTGAAGA-3′	5′-TTATTGGCACTATTGCAACCGCCC-3′
	*Br*	5′-TCTGTGACTCGGTGACATTTGCGA-3′	5′-TTACTAGACCGCTTGCCGGATTGT-3′
**Control**	*Rp49*	5′-AAGAAGCGCACCAAGCACTTCATC-3′	5′-TCTGTTGTCGATACCCTTGGGCTT-3′

### Minimum Weight to Pupariate

Larvae were cultured at 18°C. First-instar larvae were collected 48 hours after egg laying and were transferred in batches of 30 to vials containing fresh cornmeal-yeast-agar medium. At the designated time, larvae were weighed in batches of 3–5 and transferred to a 35×10 mm plate filled with either grape-juice agar (fed) or 2% agar in water (starved). Pupariation was scored in 12 hour intervals for fed and starved animals.

### Cell Ablation Strategy

Cell ablation (Figure 1) was induced essentially as described by Smith-Bolton et al (2007) with the following modifications*: w^1118^;+;+* or w*; P{Sgs3-GFP}3 females were crossed to w*;+;rn-GAL4,UAS-egr,tubGAL80^ts^/TM6Tb, tubGAL80 males [21]. Flies were conditioned for two days on fresh yeast paste and embryos were collected at room temperature in four hour intervals on grape juice agar supplemented with a small amount of fresh yeast paste. Plates were incubated at 18°C. First-instar larvae (48 hours after egg laying) were transferred in groups of 30 larvae to a vial containing standard cornmeal-yeast-agar medium. Larvae were allowed to develop at 18°C until the designated time for ablation induction. At the designated time vials were transferred to 30°C for 40 hours, returned to 18°C and monitored daily to document the time to Sgs3GFP expression, pupariation or eclosion. Ablating animals were w*;+;rn-GAL4,UAS-egr,tubGAL80^ts^/P{Sgs3GFP}3 or w*;+;rn-GAL4,UAS-egr,tubGAL80^ts^/+ (collectively referred to as Ablating). Mock-ablated discs were the siblings of the ablating animals which were w*;+;TM6Tb, tubGAL80/P{Sgs3GFP}3 or w*;+;rn-GAL4,TM6Tb, tubGAL80/+ (collectively referred to as Non-Ablating).

### Larval Collection

Cell ablation was induced at 170 hours AEL as described (Cell Ablation Strategy; Figure 1A). Larvae were collected at the following time points: (T_0_) just before induction of cell ablation (170 hrs AEL), (T_1_) half-way through cell ablation (190 hrs AEL), (T_2_) immediately after cell ablation (210 hrs AEL), and (T_3_) 24 hours after cell ablation (234 Hrs AEL). For qRT-PCR, three sets of larvae (five larvae/set) were collected for each genotype (Ablating: *w^1118^;+;rnGAL4,UAS-egr,tubGAL80^ts^/+* and Non-ablating: *w^1118^;+;TM6BTb, tubGAL80/+*) at each time point. For ecdysteroid titer measurements, two sets of larvae (15 larvae/set) were collected for each genotype (Ablating/Non-Ablating) at each time point (T_0_–T_3_). Larvae were flash frozen in liquid nitrogen and stored at −80°C for future use.

### Ecdysteroid Titer Measurements

Ecdysteroid levels were quantified via competitive Enzyme Immunoassay (EIA) (Cayman Chemicals, Inc., USA) [76], [77] using 20E (Sigma) and 20E acetylcholinesterase (Cayman Chemicals, Inc., USA) as the standard and enzymatic tracer, respectively. The antiserum detects ecdysone, 20-hydroxyecdysone and other ecdysteroid metabolites including 2-deoxy-20-hydroxyecdysone and 2-deoxyecdysone [76], [77], [78]. The standard curve was obtained with 20E (Sigma-Aldrich, USA) and results are expressed as 20E equivalents. For sample preparation, 15 staged larvae were weighed and preserved in 600 µl of methanol. Prior to the assay, samples were homogenized and centrifuged (10 minutes at 18,000×g) twice and the resulting methanol supernatants were combined and dried. Samples were resuspended in 100 µl of enzyme immunoassay (EIA) buffer (0.4 M NaCl, 1 mM EDTA, 0.1% BSA in 0.1 M phosphate buffer). Ellmann reagent (Cayman Chemicals, Inc., USA) was used for the chromogenic reaction and absorbance was read at 415 nm. All assays were performed in triplicate.

### Real-Time PCR

Larvae were homogenized in Trizol reagent (Invitrogen). RNA concentration was determined by spectrophotometric analysis. RNA was flash frozen in liquid nitrogen and stored at −80°C. Reverse transcription was carried out using qScript cDNA Supermix (VWR/Quanta). First Strand synthesis reactions were incubated as follows: 5 minutes [25°C]; 30 minutes [42°C]; 5 minutes [85°C]; hold [4°C]. cDNA was diluted 1∶5 and 5 µl was used for each qRT-PCR reaction. qRT-PCR was performed using PerfeCTa SYBR Green FastMix (VWR/Quanta). Reactions were incubated in a real time thermal detection system (Stratagene MX3000P) as follows: 95°C [2 minutes]; 40 cycles [95°C (1 second); 60°C (30 seconds)]. Fluorescence intensity was recorded at the end of each elongation phase. A dissociation curve was added to the end of the thermal cycle program. Results were analyzed by using the MxPro qPCR Software version 4.1 (Stratagene) and relative expression levels were normalized to mRNA for *ribosomal protein L32* (*RpL32/Rp49*). Primers used for qRT-PCR are shown in Table 1. Statistical analyses were performed using the nonparametric Mann-Whitney *U* test.

## Supporting Information

Figure S1 **Wing Phenotypes Following Cell Ablation.** All flies shown were heat treated at 198 hours AEL to induce cell ablation. (A) Control (Non-Ablating) fly. (B–E) Flies from the Ablating genotype representing the range of wing phenotypes obtained following cell ablation at 198 hours AEL.
(TIF)

Figure S2 **qRT-PCR Analysis of Ecdysteroidogenic Enzymes Following Cell Ablation for time points T_0_–T_3_.**
(TIF)

Figure S3 **qRT-PCR Analysis of Ecdysteroidogenic Genes and Ecdysone Oxidase Following Cell Ablation for time points T_0_–T_3_.**
(TIF)

Figure S4 **qRT-PCR Analysis of Ecdysone Receptor Components Following Cell Ablation for time points T_0_–T_3_.** Two primers sets (EcR-RA and EcR-RE) that each amplify a region common to all EcR isoforms were utilized to characterize the EcR response.
(TIF)

Figure S5 **qRT-PCR Analysis of Ecdysone Response Genes Following Cell Ablation for** **time points T_0_–T_3_.**
(TIF)
