# Supplementary figures and images for: Tissue Damage Disrupts Developmental Progression and Ecdysteroid Biosynthesis in Drosophila
Source: PLoS One. 2012 Nov 13;7(11):e49105. doi: 10.1371/journal.pone.0049105 (PMC3496736; doi:10.1371/journal.pone.0049105)

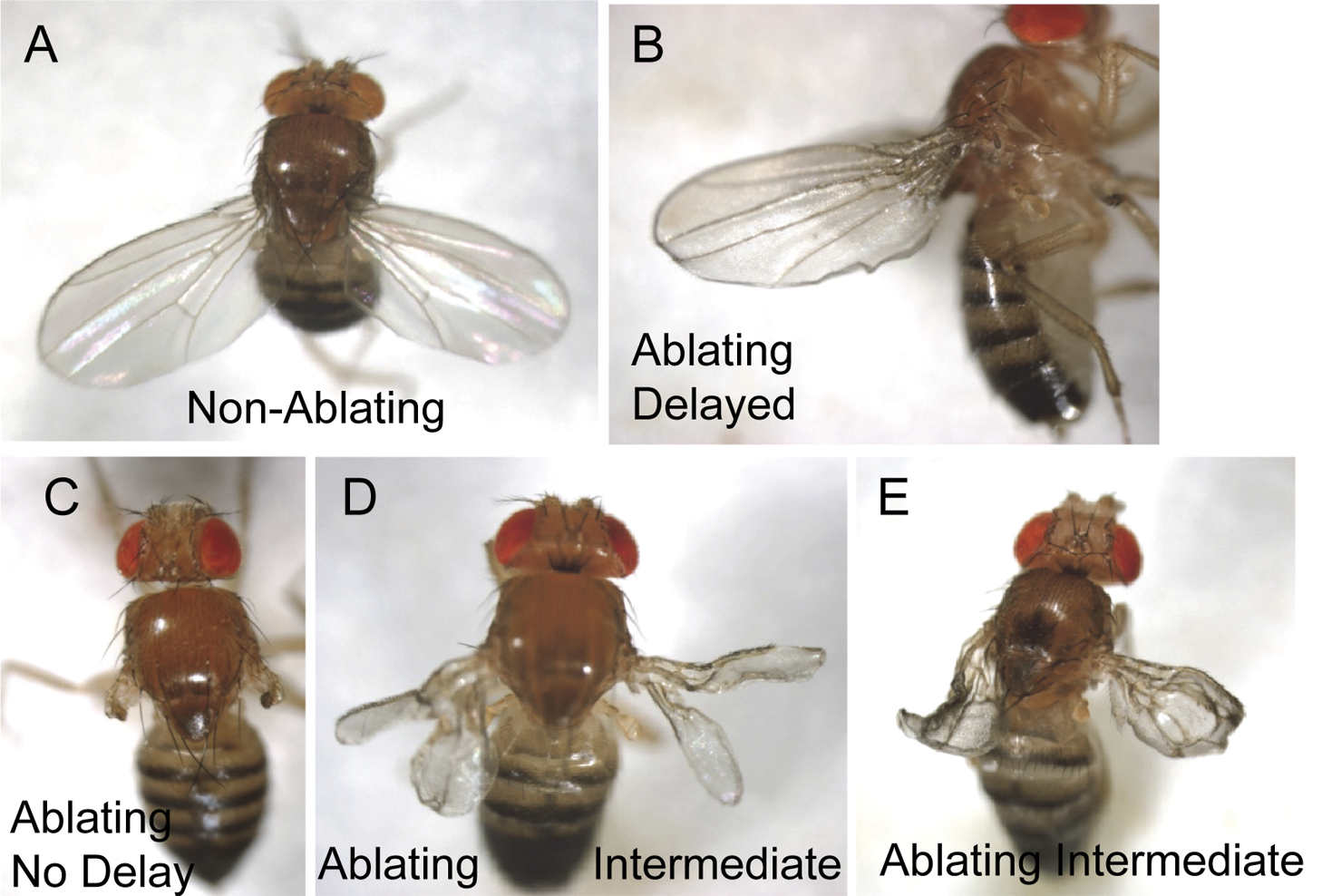

Supplement: Figure S1 — Wing Phenotypes Following Cell Ablation. All flies shown were heat treated at 198 hours AEL to induce cell ablation. (A) Control (Non-Ablating) fly. (B–E) Flies from the Ablating genotype representing the range of wing phenotypes obtained following cell ablation at 198 hours AEL. (TIF) [file pone.0049105.s001.tif]

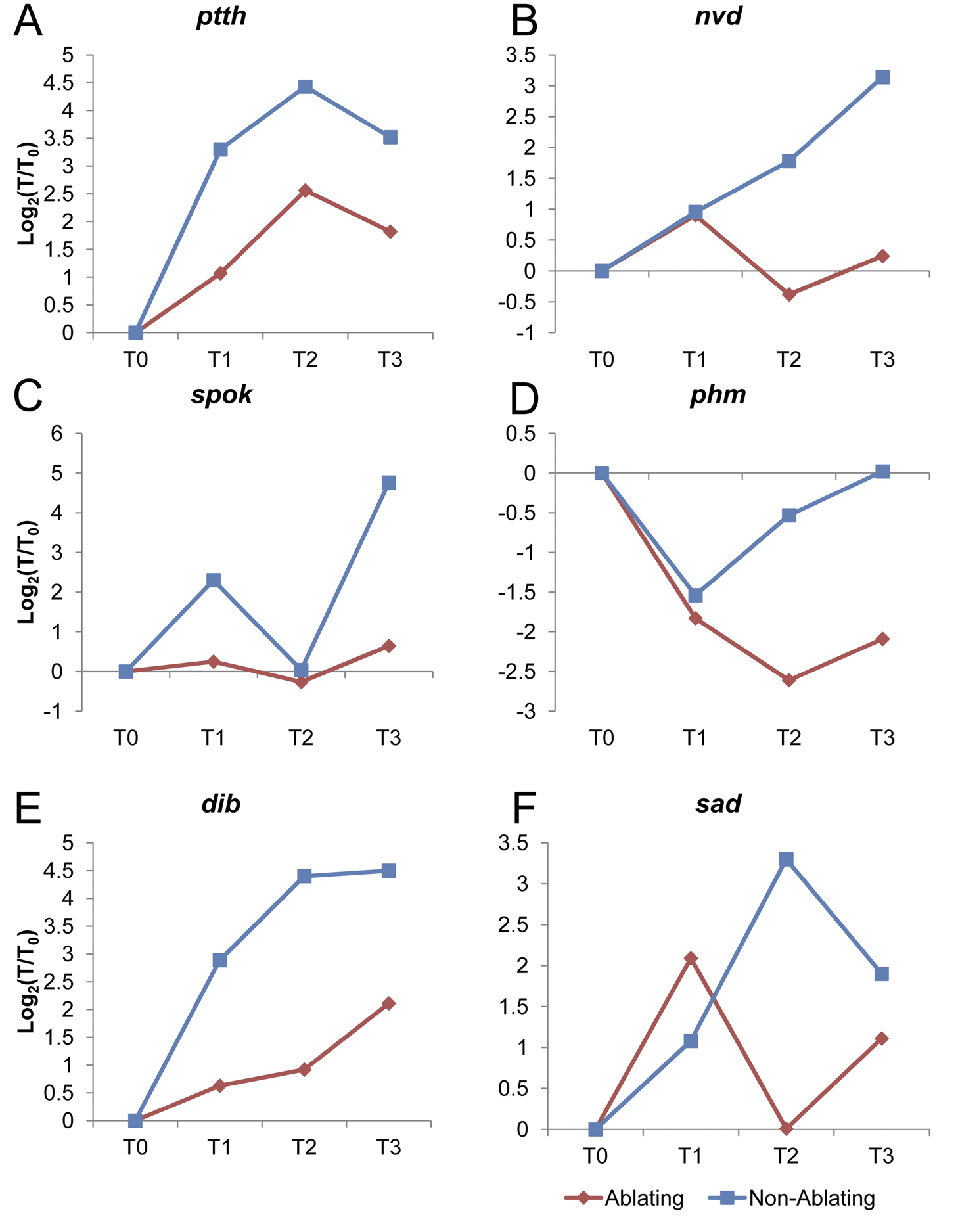

Supplement: Figure S2 — qRT-PCR Analysis of Ecdysteroidogenic Enzymes Following Cell Ablation for time points T0–T3. (TIF) [file pone.0049105.s002.tif]

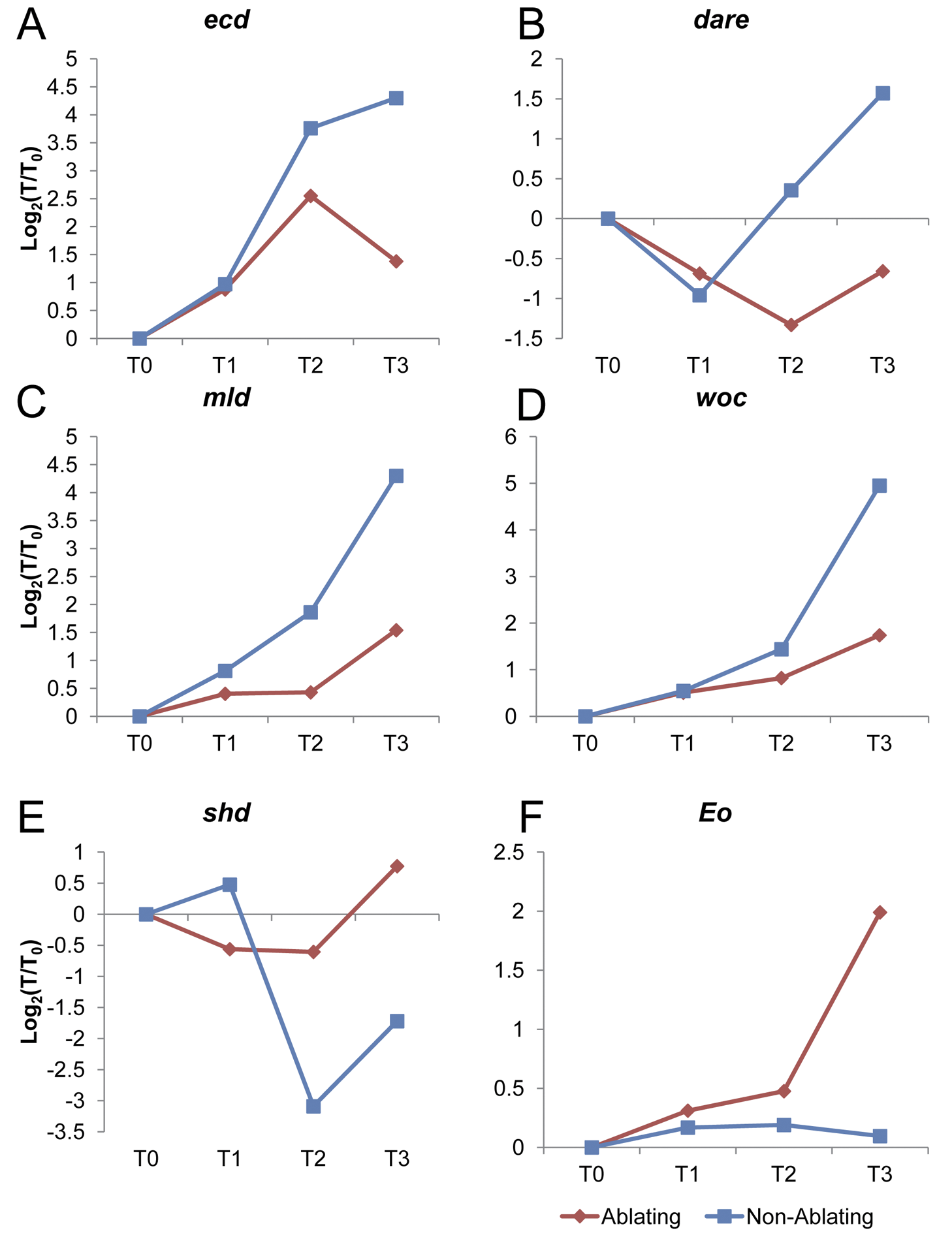

Supplement: Figure S3 — qRT-PCR Analysis of Ecdysteroidogenic Genes and Ecdysone Oxidase Following Cell Ablation for time points T0–T3. (TIF) [file pone.0049105.s003.tif]

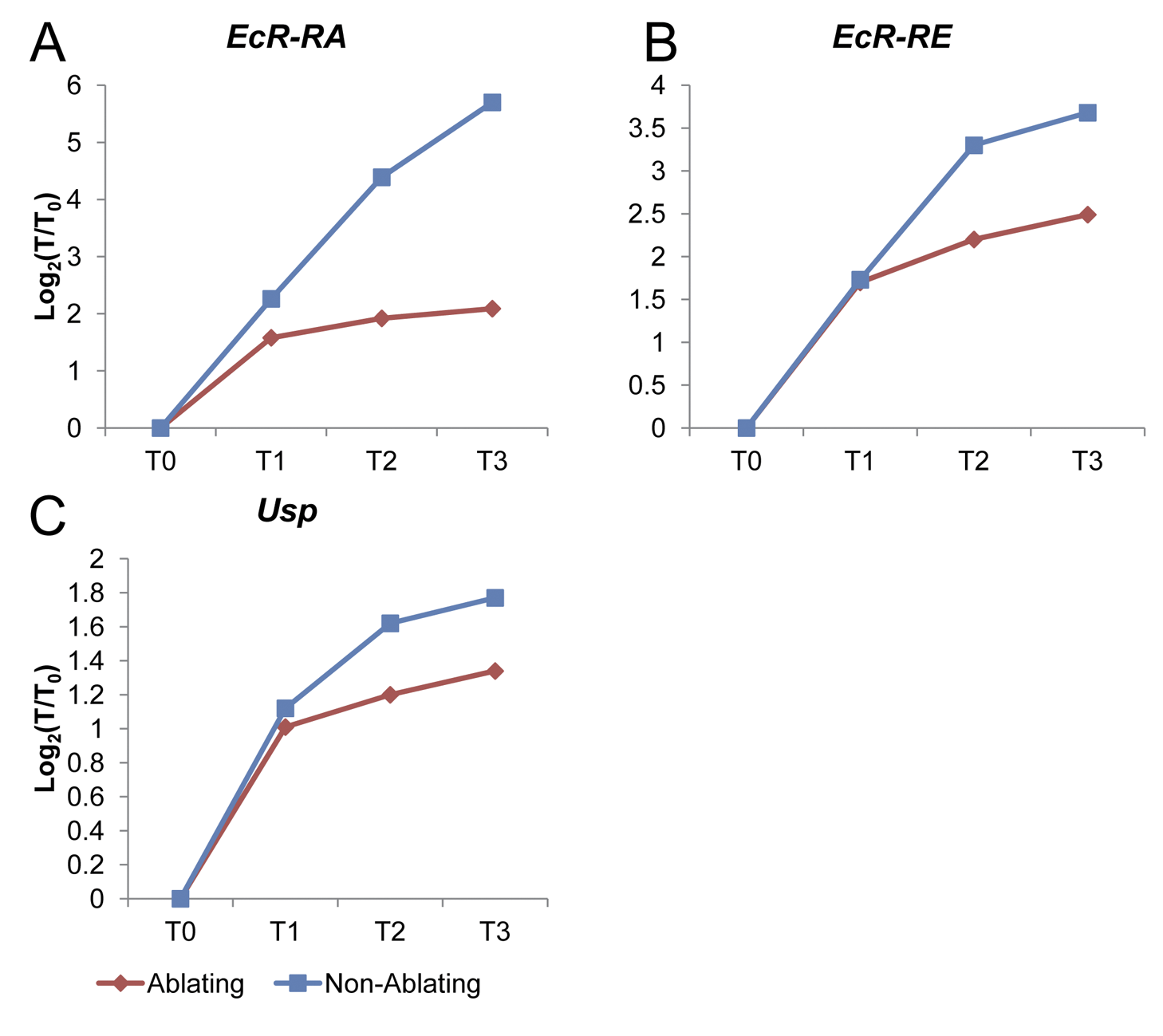

Supplement: Figure S4 — qRT-PCR Analysis of Ecdysone Receptor Components Following Cell Ablation for time points T0–T3. Two primers sets (EcR-RA and EcR-RE) that each amplify a region common to all EcR isoforms were utilized to characterize the EcR response. (TIF) [file pone.0049105.s004.tif]

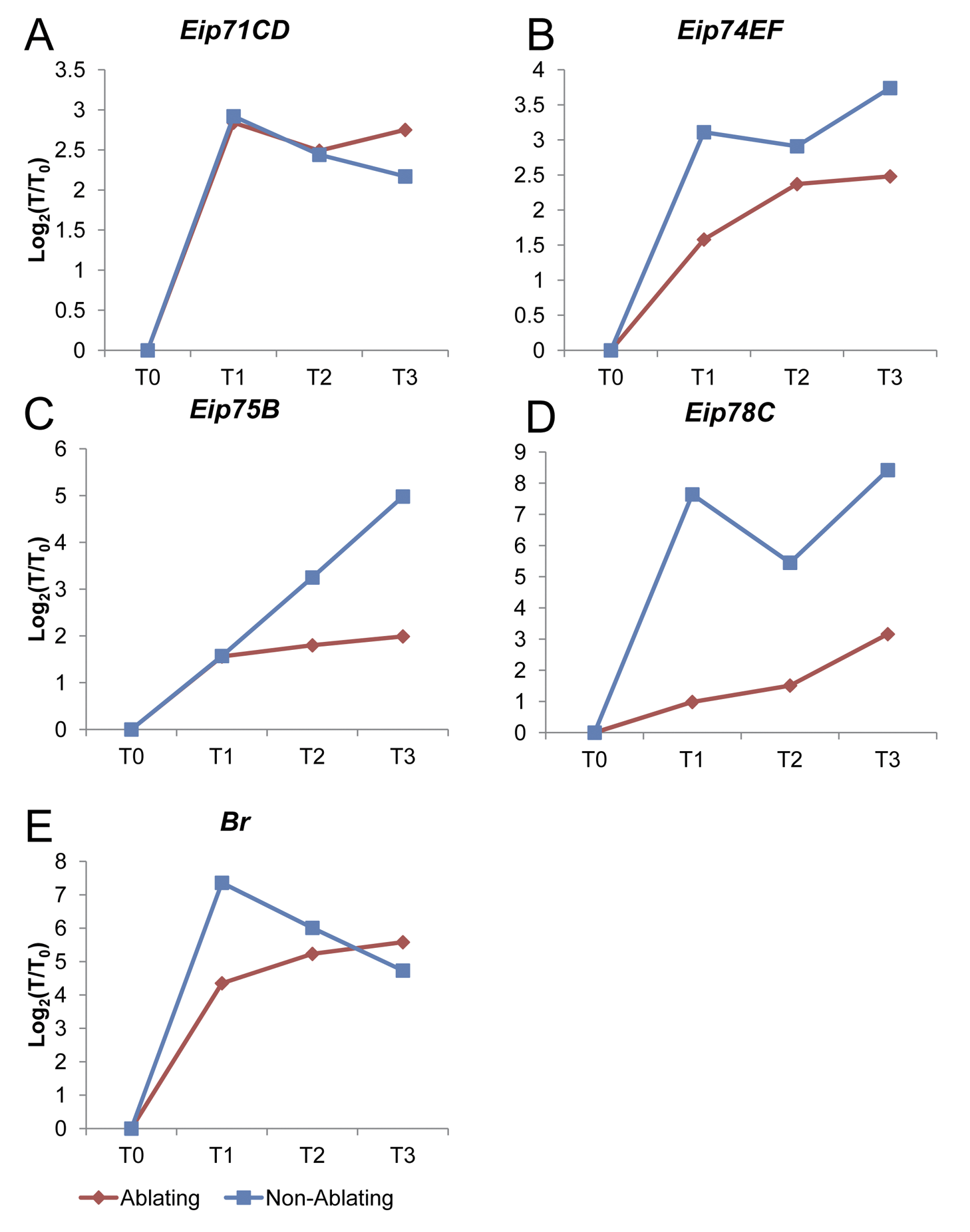

Supplement: Figure S5 — qRT-PCR Analysis of Ecdysone Response Genes Following Cell Ablation for time points T0–T3. (TIF) [file pone.0049105.s005.tif]
